# A Prior History of Cryptozoospermia Is Associated with a Significantly Higher Chance of a Successful Microdissection Testicular Sperm Extraction Compared to Non-Obstructive Azoospermia

**DOI:** 10.3390/jcm12237255

**Published:** 2023-11-23

**Authors:** James Wren, Matthew Hudnall, Minh Pham, Anne L. Darves-Bornoz, Joshua A. Halpern, Nelson E. Bennett, Robert E. Brannigan, Matthias D. Hofer

**Affiliations:** Department of Urology, Northwestern University Feinberg School of Medicine, 676 North St. Clare St., Arkes Pavilion 23-025, Chicago, IL 60611, USAminh@ad.unc.edu (M.P.); joshua.halpern@northwestern.edu (J.A.H.); nelson.bennett@nm.org (N.E.B.); r-brannigan@northwestern.edu (R.E.B.); matthias.hofer@urologysa.com (M.D.H.)

**Keywords:** cryptozoospermia, azoospermia, microdissection testicular sperm extraction

## Abstract

Background: Our study sought to evaluate the rates of successful sperm retrieval following microdissection testicular sperm extraction (mTESE) in patients with a prior history of cryptozoospermia, compared to patients with non-obstructive azoospermia (NOA). Methods: A retrospective chart analysis evaluating all mTESE procedures was performed from January 2004 to August 2018. Inclusion criteria involved all males >18 years of age with a diagnosis of cryptozoospermia and/or NOA that underwent a mTESE. The patient’s genetic profile, hormonal profile, semen analysis, testicular volumes, pathology and comorbidities were analyzed. Results: We identified 40 patients with cryptozoospermia and 221 patients with NOA. Successful mTESE occurred in 34/40 (85%) cryptozoospermic males compared to 104/221 (48%) NOA males (*p* < 0.001). In univariate and multivariate analyses, patients with cryptozoospermia were more likely to undergo a successful mTESE than patients with NOA (OR 5.56 [1.79–17.29], *p* = 0.003; OR 5.41 [1.94–15.08], *p* = 0.0013), respectively. Factors that were associated with a statistically significant lower chance of successful mTESE included Sertoli-cell only pathology, pre-operative testosterone < 300 ng/dL and FSH > 7.6 mIU/mL. Conclusion: Despite patients with a history of cryptozoospermia having a significantly higher chance of a successful sperm retrieval than patients with NOA, couples should be counselled on the possibility of an unsuccessful sperm extraction, in order to optimize the pre-operative IVF planning and to manage operative expectations.

## 1. Introduction

One in six couples ultimately require an infertility assessment, with 50% of couples having a male factor identified that needs to be optimized [1,2,3]. An abnormal semen analysis can be found in 52% of men presenting for a fertility assessment, with the most common abnormalities including a low sperm concentration (oligospermia), low numbers of motile sperm (asthenozoospermia), sperm with abnormal morphology (teratozoospermia), or a combination of the above [4,5]. Cryptozoospermia is the absence of sperm on a fresh ejaculated preparation that ultimately reveals spermatozoa in the centrifuged pellet [6]. Cryptozoospermia is found in 8.5% of males undergoing an infertility work up and reflects a form of testicular failure where only small foci of active spermatogenesis persist within the testicles [7,8]. Although uncommon, cryptozoospermia has been associated with genetic, hormonal, infectious and malignant etiologies [8]. Azoospermia, the absence of sperm in the fresh preparation and centrifuged pellet, is found in 4–15% of males undergoing fertility evaluation [4,9,10]. Azoospermia is divided into non-obstructive azoospermia (NOA) and obstructive azoospermia (OA) [11,12]. NOA results from spermatogenic failure and accounts for 60% of all patients with azoospermia. OA accounts for the remaining 40% of patients with azoospermia and results from excurrent ductal obstruction (rete testes, efferent ducts, epididymis, vas deferens and ejaculatory duct), in the setting of normal spermatogenesis.

Patients with cryptozoospermia may require a microdissection testicular sperm extraction (mTESE) due to one of three reasons. First, men may progress from cryptozoospermia to azoospermia on a subsequent semen analysis. Second, some men may present with alternating cryptozoospermia and azoospermia, and the uncertainty as to whether sperm will be present in a fresh ejaculated sample on the day of in vitro fertilization (IVF) may necessitate mTESE to ensure that sperm are available at the time of egg retrieval in the female partner [13]. The third, which is more controversial, occurs in couples with recurrent IVF failure using ejaculated sperm in the setting of cryptozoospermia [13,14,15,16,17].

Sperm retrieval rates (SRRs) from microdissection testicular sperm extraction in patients with non-obstructive azoospermia (NOA) have been extensively studied, with SRRs ranging from 23% to 72% [18,19,20,21]. However, limited evidence exists to counsel patients with cryptozoospermia on their chance of a successful sperm retrieval during mTESE [22]. A misconception exists amongst patients that a prior semen analysis demonstrating cryptozoospermia will inevitably result in a successful sperm retrieval. This is inaccurate and establishing SRRs in cryptozoospermic patients will assist in a couple’s operative expectations and pre-operative decision making, especially if patients are looking to proceed with a fresh versus frozen mTESE. In cases where there exists a high chance of an unsuccessful fresh mTESE, the couple’s IVF management may change, with some couples electing to organize donor sperm as a backup plan in anticipation of possible unsuccessful mTESE. Therefore, our study sought to assess the rates of successful sperm retrieval in patients with a prior history of cryptozoospermia compared to patients with NOA, in order to help physicians counsel patients on their likelihood of a successful mTESE.

## 2. Methods

### 2.1. Patient Cohort

Following institutional review board (IRB) approval, we queried the Northwestern Enterprise Data Warehouse to identify all mTESE procedures performed for non-obstructive azoospermia and cryptozoospermia from January 2004 to August 2018. We included all males >18 years of age with a diagnosis of cryptozoospermia and/or non-obstructive azoospermia who underwent a mTESE. Patients with one or more semen analyses showing cryptozoospermia were assigned to the cryptozoospermia group, while patients with azoospermia in all semen analyses were assigned to the azoospermia group. Exclusion criteria included those with a previous diagnosis of oligospermia or less than two semen analyses. Patients were also excluded if there was missing genetic testing, hormonal profile or testicular histopathology. A retrospective chart analysis was performed, and descriptive statistics were utilized to characterize patient demographics, comorbidities (varicocele, history of cryptorchidism, history of prior or current malignancy, history of chemotherapy or genetic diagnosis), hormonal profile (serum follicle stimulation hormone [FSH], serum total testosterone), semen parameters and testis histopathology. Genetic testing in the setting of NOA and cryptozoospermia included karyotype and Y-chromosomal microdeletion. A successful sperm retrieval was defined as the presence of intraoperative sperm found at the time of microdissection testicular sperm extraction.

### 2.2. Surgical Procedure and Tissue Processing

The microdissection testicular sperm extraction procedure was performed under general anesthesia as described by Schlegel et al. [23]. After induction of general anesthesia, a unilateral horizontal hemi-scrotal incision was used to carry the dissection through the subcutaneous tissues and deliver the testis with the tunica vaginalis intact. After opening the tunica vaginalis, the operating microscope was brought into the field and an equatorial incision was made at the mid-pole of the testis. The testis was bi-valved, and careful, meticulous microdissection was then undertaken with systematic mapping of the upper and lower pole of the testicle. Microscopic examination optimized the attempted identification and extraction of large-diameter tubules with opalescent appearance. Tissue was processed manually on the operative table, and intra-operative cytologic assessment was performed with phase-contrast microscopy by an embryologist. In locations of the testicle where systematic mapping had identified sperm, additional tissue extraction occurred. All specimens were sent to the andrology laboratory for further manual processing and enzymatic treatment. A formal testis biopsy was sent for histopathological evaluation in all cases. In cases where no sperm could be identified intra-operatively after unilateral dissection, bilateral microdissection testicular sperm extraction was performed via a second contralateral horizontal hemi-scrotal incision. Upon completion of the tissue sampling and extraction, hemostasis was achieved with bipolar electrocautery and the tunica albuginea was closed with 4-0 absorbable sutures. The tunica vaginalis was closed with 3-0 absorbable sutures and the skin with 4-absorable sutures.

### 2.3. Primary Outcome and Statistical Analysis

The primary outcome was successful sperm retrieval. Descriptive statistics summarized sample demographic characteristics and clinical measures. Analyses employed Pearson’s Chi-Squared tests to assess the association between retrieval success and categorical measures. Continuous variables were compared across groups using one-way ANOVA for normally distributed data and Kruskal–Wallis tests when statistical assumptions (i.e., normality) were questionable. We also performed multiple logistic regressions on retrieval outcomes adjusting for the potential confounding effects. All analyses assumed a 5% level of significance. Analyses were performed using R: a language and environment for statistical computing (R Foundation for Statistical Computing, Vienna, Austria; Version 3.3.2).

## 3. Results

**Figure 1:**
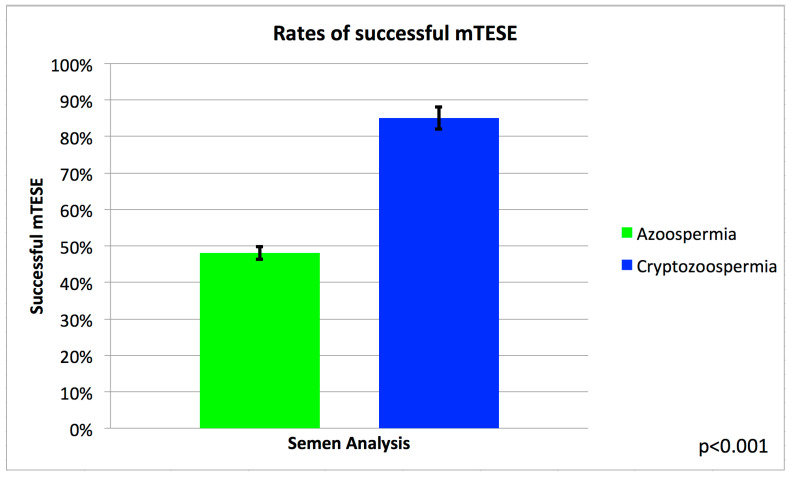
A bar graph demonstrating the rates of successful micro-testicular sperm extraction (mTESE) between males with azoospermia compared to cryptozoospermia.

**Figure 2:**
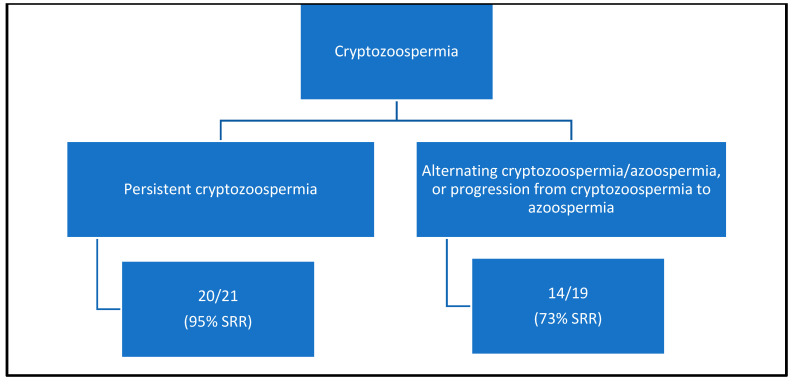
Flow diagram of the chance of successful sperm retrieval (SSR) following mTESE.

We identified 40 patients with cryptozoospermia and 221 patients with NOA. Successful mTESE occurred in 34/40 (85%) cryptozoospermic males compared to 104/221 (48%) males with NOA (Figure 1). Of the 40 cryptozoospermic patients, a mTESE was performed in 19 males (47%) due to their progression from cryptozoospermia to NOA or alternating cryptozoospermia/NOA (Figure 2). In 21 males (53%), a mTESE was performed due to prior failed IVF attempts using a fresh ejaculated cryptozoospermic sample, or following recommendations from the reproductive endocrinologist to obtain testicular sperm for IVF/ICSI. In the univariate analysis (Table 1, Table 2 and Table 3), cryptozoospermia was associated with a higher SRR than patients with NOA (OR 5.56 [1.79–17.29], *p* = 0.003). This persisted in the multivariate analysis (OR 5.41 [1.94–15.08], *p* = 0.0013) (Table 4). In the univariate analysis, other factors associated with a statistically significant successful sperm retrieval included pathology of hypospermatogenesis and active spermatogenesis, pre-operative testosterone > 300 ng/dL and FSH < 7.6 mIU/mL. Factors that were associated with a statistically significant lower chance of successful microdissection testicular sperm extraction included pathology of Sertoli cell only, pre-operative serum total testosterone < 300 ng/dL and FSH > 7.6 mIU/mL (Table 2 and Table 3). In the multivariate analysis, factors that remained statistically significant included cryptozoospermia, hypospermatogenesis, Sertoli cell only and a serum total testosterone < 300 ng/dL (Table 4). All patients with serum testosterone levels < 300 ng/dL were found to have elevated luteinizing hormone levels (>10 ng/dL), consistent with primary testicular failure. The mean time from the last semen analysis to the mTESE in the cryptozoospermia group was 90.4 days. Patients with ‘persistent cryptozoospermia’ were found to have a higher SRR (95%) when compared to alternating cryptozoospermia/azoospermia and cryptozoospermia who progressed to azoospermia (73%) (Figure 2).

## 4. Discussion

Advances in assisted reproductive technology (ART) have permitted the use of cryptozoospermic sperm for IVF or IVF/ICSI [24,25]. However, circumstances may arise where patients with cryptozoospermia require a microdissection testicular sperm extraction in order to provide sperm for IVF [26]. Limited evidence exists to be able to counsel cryptozoospermic patients on the chance of a successful sperm retrieval during a mTESE. A previous small study found that men with cryptozoospermia who underwent a mTESE had a 92% chance of a successful intraoperative mTESE, with 87.5% of patients only requiring a unilateral mTESE [22]. However, the study did not clarify if patients had persistent cryptozoospermia on repeat semen analysis, or if prior or subsequent semen analyses were consistent with oligospermia or non-obstructive azoospermia. There was also no mention of the number of patients with alternating cryptozoospermia/non-obstructive azoospermia. Our study found that patients with a history of cryptozoospermia had an 85% chance of having a successful microdissection testicular sperm extraction. This increased to 95% in patients with persistent cryptozoospermia on repeat semen analysis but decreased to 73% in patients who progressed from cryptozoospermia to non-obstructive azoospermia, or in patients with alternating azoospermia/cryptozoospermia. There was also an associated higher retrieval rate of 91% (21/23) in patients with idiopathic cryptozoospermia, compared to 76% (12/17) if there was an underlying risk factor identified, such as a history of cryptorchidism, testicular cancer, prior chemotherapy or surgical correction of a varicocele. Of the 21 successful idiopathic cryptozoospermic mTESEs, 61% (14/23) underwent a unilateral mTESE, while 39% (9/23) required a bilateral approach. Overall, we did not find that cryptozoospermia was associated with a lower chance of requiring a unilateral procedure when compared to patients with non-obstructive azoospermia (52% and 48%, respectively) (*p* = 0.6).

The presence or history of cryptozoospermia may provide couples and physicians with a false sense of security when contemplating the surgical sperm retrieval approach. A decision to attempt a less invasive form of surgical sperm retrieval than a mTESE, such as a testicular sperm aspiration (TESA), should be avoided. A TESA yields inferior sperm retrieval rates of 25% in patients with a history of cryptozoospermia, when compared to the microdissection testicular sperm extraction approach [27]. More recently, a ‘mini-incision’ microdissection testicular sperm extraction approach in the setting of cryptozoospermia has been reported [28]. This approach uses a smaller 1 cm initial tunica albuginea incision which can be extended to the standard incision template if sperm are not found in the testicular tissue assessment. Success was reported if ≥5 spermatozoa were found on intra-operative cytologic assessment with phase-contrast microscopy. A success rate of 58% was reported with the ‘mini-incision’ approach, that ultimately increased to 89% when the incision was extended to the standard template [28]. Although a mini-approach may be applicable to specific clinical scenarios, we advocate for the standard microdissection testicular sperm extraction template in order to optimize sperm retrieval rates and sperm yield [29]. The standard mTESE template offers the advantage of a higher sperm yield, thereby reducing the need for a repeat mTESE where retrieval rates are potentially lower as a result of ischemia, atrophy, fibrosis or disease progression [30].

Previous studies examining patients with non-obstructive azoospermia have identified an association between pre-operative elevated FSH levels and Sertoli-cell-only histology in testicular biopsy following mTESE [31,32,33,34,35]. We found that a significant proportion of patients with cryptozoospermia had a final histopathology consistent with hypospermatogenesis. Our analysis to assess if cryptozoospermia could be used as a surrogate for hypospermatogenesis histopathology did not, however, reach statistical significance. Our study found that an unsuccessful mTESE was associated with serum testosterone < 300 ng/dL, elevated FSH and Sertoli-cell-only histology, which is consistent with other studies [31,32,34,35].

Cryptozoospermia has been associated with genetic, hormonal, infectious and malignant etiologies [8]. Genetic conditions include Klinefelter syndrome and Y-chromosomal microdeletion. Hypogonadism and orchitis are previously reported endocrine and infectious causes [17]. Malignancy-associated cryptozoospermia or cryptozoospermia resulting from gonadotoxic chemotherapy have been observed; however, the majority of the patients in our study, 23 (57%), were idiopathic [8]. In addition to the previously mentioned etiologies, we identified cryptozoospermia in patients with a history of cryptorchidism and prior microsurgical varicocelectomy.

In vitro fertilization with intracytoplasmic sperm injection (IVF/ICSI) was first reported in 1992 and is the mainstay of treatment for couples with cryptozoospermia [36]. While ejaculated sperm is more easily obtained and does not require an invasive procedure, it has potential shortcomings. Controversy surrounds the use of ejaculated sperm versus testicular sperm in cryptozoospermic males due to potentially higher DNA fragmentation, which has been associated with worse ART outcomes. Elevated DNA fragmentation results from oxidative stress as sperm transit through the male genital tract. Elevated DNA fragmentation index has been found to negatively influence the probability of pregnancy, either naturally or via ART [37,38]. An increased DNA fragmentation index is associated with increased miscarriage rates due to the late paternal effects that DNA fragmentation has on embryogenesis [39,40]. Several etiologies have been associated with increased oxidative stress and include obesity, alcohol, smoking, infection, increase paternal age, hyperthermia and chemotherapy [41]. In contrast, testicular sperm obtained from a microdissection testicular sperm extraction may have lower DNA fragmentation levels; however, testicular sperm is generally associated with higher sperm aneuploidy [42,43]. Sperm aneuploidy has been shown to contribute to early paternal effects on embryogenesis which contribute to lower cleavage rates and blastocyst formation rates [44]. Three meta-analyses that addressed the issue of ejaculated versus testicular sperm resulted in conflicting results [13,45,46]. Although one showed no difference in fertilization and pregnancy rates, others demonstrated that testicular sperm was associated with improved implantation and pregnancy rates, along with higher live birth rates per embryo transfer [13,45,46]. More recently, there is evidence that in couples with multiple failed IVF/ICSI cycles using ejaculated cryptozoospermic sperm, utilizing testicular sperm following a microdissection testicular sperm extraction is associated with higher pregnancy and live birth rates [17]. The use of testicular sperm in this clinical scenario may offer couples a viable therapeutic option. In our study, 21 patients (53%) underwent IVF/ICSI using testicular sperm due to multiple failed IVF/ICSI attempts using ejaculated sperm. The miscarriage, biochemical pregnancy, clinical pregnancy and live birth rates were, however, not available.

In our study, the overall success rate for a mTESE was significantly higher in patients with cryptozoospermia compared to those with NOA. Despite this, it is important to counsel patients with cryptozoospermia that there remains an overall 15% chance of an unsuccessful mTESE, even though sperm had previously been seen in the ejaculate (mean time of 90.1 days prior). In patients with persistent cryptozoospermia, there is a greater chance of a successful retrieval (95%). However, patients with alternating cryptozoospermia/azoospermia or cryptozoospermia that progressed to azoospermia had a significantly lower chance of successful retrieval (73%). The category that the male partner falls into may influence a couple’s pre-operative decision to proceed with back-up donor sperm, in the event of an unsuccessful fresh mTESE, or instead elect to undergo a frozen mTESE. Additionally, these findings may assist in pre-operative counseling and planning, specifically when considering arranging donor sperm as a backup plan in anticipation of a possible unsuccessful mTESE. A commonly encountered preoperative clinical expectation among couples whose male partner has cryptozoospermia is that sperm will inevitably be found intraoperatively because sperm was previously seen in the ejaculate. However, as our study demonstrates, an unsuccessful mTESE can occur in 5–27% of patients, and these preoperative expectations need to be established by the surgeon.

In the present study, the time from the semen analysis to mTESE was 90.1 days. In patients who have progressed from cryptozoospermia to NOA without prior attempts at IVF/ICSI, there may be a benefit derived from a semen analysis on the day of surgery, as 9.5% of patients with NOA have been found to have sperm in the ejaculate [47]. If sperm are identified on the day of the surgery (the day before the oocyte retrieval), the sperm could be used for IVF/ICSI in order to avoid surgery or instead be utilized as a backup in the event of an unsuccessful mTESE.

Hormonal biomarkers such as FSH, total serum testosterone, estradiol and anti-mullerian hormone (AMH) have been previously assessed as possible predictors of successful mTESE. Historically, elevated FSH levels (>7.6 mIU/mL) were seen as a surrogate for spermatogenic failure and a predictor of mTESE outcome in patients with NOA [48]. Consistent with prior studies, we found that elevated FSH (>7.6 mIU/mL) was associated with unsuccessful mTESE, though this should not dissuade couples from pursuing mTESE, given prior evidence showing that elevated FSH does not preclude a successful mTESE [49]. Also consistent with prior studies, we found that total serum testosterone levels < 300 ng/dL were associated with lower SRRs. However, controversy exists as to the importance of baseline total testosterone levels, with evidence suggesting that the total testosterone level does not influence the rates of successful sperm retrieval [50]. Serum AMH levels were not assessed in our study as AMH is not considered part of a patients routine hormonal work up; however, a recent multi-center study of patients with idiopathic NOA raises the possibility that in this select patient population, a low AMH (<4 ng/dL) may serve as a predictor of a successful mTESE [51]. Further studies are needed to investigate the role of AMH in patients with idiopathic NOA as a predictor of a successful mTESE.

Despite being the largest study to date to assess the success rates of mTESE in patients with cryptozoospermia, limitations of the study include the relatively small number of patients, retrospective nature and unavailable IVF/ICSI outcomes.

Future directions to assist in optimizing the counselling of patients with a history of cryptozoospermia would involve additional studies that investigate each of the individual categories of patients (persistent cryptozoospermia vs. alternating cryptozoospermia/NOA vs. progression to NOA) in relation to their IVF/ICSI outcomes. If a difference is observed between the groups in relation to sperm retrieval, clinical pregnancy and live birth rates, this may influence a couple’s decision to proceed directly to mTESE, or instead schedule donor backup sperm, or proceed directly to IVF with donor sperm.

## 5. Conclusions

Patients with a history of cryptozoospermia were found to have a significantly higher chance of a successful microdissection testicular sperm extraction than patients with non-obstructive azoospermia. However, 15% of cryptozoospermic patients did not have successful sperm retrieval with mTESE. Couples should be counselled on the possibility of an unsuccessful sperm extraction in order to optimize pre-operative IVF planning and to manage a couple’s operative expectations.

## Figures and Tables

**Table 1:** Clinical parameters associated with successful sperm retrieval.

	Retrieval Successful		Retrieval Not Successful		Total		
	*n*	%	*n*	%	*n*	%	*p* Value
**Age**							0.3346
Median	34		34		34		
IQR	(31–40)		(31–36)		(31–38)		
**Semen Analysis**							<0.0001
Cryptozoospermia	34	(24.6)	6	(4.9)	40	(15.3)	
Azoospermia	104	(75.4)	117	(95.1)	221	(84.7)	
**Genetic Diagnosis**							0.3975
Yes	12	(12.2)	16	(16.5)	28	(14.4)	
No	86	(87.8)	81	(83.5)	167	(85.6)	
**Varicocele**							0.7010
Yes	19	(13.8)	19	(15.4)	38	(14.6)	
No	119	(86.2)	104	(84.6)	223	(85.4)	
**Cryptorchidism**							0.6489
Yes	11	(8.0)	8	(6.5)	19	(7.3)	
No	127	(92.0)	115	(93.5)	242	(92.7)	
**Cancer/chemotherapy**							0.9901
Yes	19	(13.8)	17	(13.8)	36	(13.8)	
No	119	(86.2)	106	(86.2)	225	(86.2)	
**Idiopathic**							0.4836
Yes	80	(58.0)	66	(53.7)	146	(55.9)	
No	58	(42.0)	57	(46.3)	115	(44.1)	
**Sertoli Cell Only**							<0.0001
Yes	29	(22.7)	90	(75.0)	119	(48.0)	
No	99	(77.3)	30	(25.0)	129	(52.0)	
**Hypospermatogenesis**							<0.0001
Yes	39	(30.5)	3	(2.5)	42	(16.9)	
No	89	(69.5)	117	(97.5)	206	(83.1)	
**Maturation Arrest**							0.1375
Yes	17	(13.3)	9	(7.5)	26	(10.5)	
No	111	(86.7)	111	(92.5)	222	(89.5)	
**Active Spermatogenesis**							<0.0001
Yes	25	(19.5)	0	(0.0)	25	(10.1)	
No	103	(80.5)	120	(100.0)	223	(89.9)	
**Tubular Atrophy**							0.1951
Yes	8	(6.3)	13	(10.8)	21	(8.5)	
No	120	(93.8)	107	(89.2)	227	(91.5)	
**Hyalinization**							0.4619
Yes	8	(6.3)	5	(4.2)	13	(5.2)	
No	120	(93.8)	115	(95.8)	235	(94.8)	
**Fibrosis**							0.4985
Yes	2	(1.6)	0	(0.0)	2	(0.8)	
No	126	(98.4)	120	(100.0)	246	(99.2)	
**Preoperative Testosterone**							0.0017
<300	25	(18.8)	43	(36.4)	68	(27.1)	
≥300	108	(81.2)	75	(63.6)	183	(72.9)	
**Preoperative FSH**							0.0002
<7.6	38	(29.5)	12	(10.2)	50	(20.2)	
≥7.6	91	(70.5)	106	(89.8)	197	(79.8)	
**Preoperative Testosterone**							0.0877
Median	375		347		364		
IQR	(321–459)		(258–467)		(293–463)		
**Preoperative FSH**							0.0008
Median	15.6		22.1		19.3		
IQR	(5.9–27.1)		(14.3–27.1)		(9.7–27.1)		
**Total Testicular Volume**							0.1608
Median	24		20		22		
IQR	(16–36)		(14–32)		(15–32)		

**Table 2:** Clinical parameters associated with successful sperm retrieval.

	Odds Ratio	95% CI	*p* Value
**Age**	1.01	(0.96–1.06)	0.6573
**Semen Analysis**			
Cryptozoospermia	5.56	(1.79–17.29)	0.003
Azoospermia	Ref	--	--
**Genetic Diagnosis**			
Yes	0.79	(0.35–1.79)	0.5726
No	Ref	--	--
**Varicocele**			
Yes	1.45	(0.63–3.3)	0.3804
No	Ref	--	--
**Cryptorchidism**			
Yes	1.1	(0.34–3.56)	0.8717
No	Ref	--	--
**Cancer/chemotherapy**			
Yes	1.11	(0.42–2.93)	0.8403
No	Ref	--	--
**Idiopathic**			
Yes	0.93	(0.52–1.68)	0.8098
No	Ref	--	--
**Sertoli Cell Only**			
Yes	0.08	(0.04–0.16)	<0.0001
No	Ref	--	--
**Hypospermatogenesis**			
Yes	24.81	(5.71–107.87)	<0.0001
No	Ref	--	--
**Maturation Arrest**			
Yes	2.08	(0.67–6.49)	0.2049
No	Ref	--	--
**Tubular Atrophy**			
Yes	0.53	(0.15–1.81)	0.3074
No	Ref	--	--
**Hyalinization**			
Yes	1.34	(0.39–4.55)	0.6425
No	Ref	--	--
**Preoperative Testosterone**			
<300	0.3	(0.15–0.61)	0.0008
≥300	Ref	--	--
**Preoperative FSH**			
<7.6	Ref	--	--
≥7.6	0.27	(0.11–0.65)	0.0032
**Preoperative Testosterone**	1	(1–1)	0.1967
**Preoperative FSH**	1	(0.99–1.01)	0.9029
**Total Testicular Volume**	1.01	(0.98–1.03)	0.7157

**Table 3:** Logistic regression with all significant variables from univariate analysis.

	Odds Ratio	95% CI	*p* Value
**Semen Analysis**			
Cryptozoospermia	5.11	(1.79–14.60)	0.0024
Azoospermia	ref	--	--
**Sertoli Cell Only**			
Yes	0.18	(0.09–0.35)	<0.0001
No	ref	--	--
**Hypospermatogenesis**			
Yes	6.16	(1.68–22.65)	0.0062
No	ref	--	--
**Preoperative Testosterone**			
<300	0.4	(0.19–0.85)	0.0165
≥300	ref	--	--
**Preoperative FSH**			
<7.6	ref	--	--
≥7.6	0.49	(0.20–1.20)	0.1204

**Table 4:** Multivariate analysis of clinical parameters associated with successful retrieval.

	Odds Ratio	95% CI	*p* Value
**Semen Analysis**			
Cryptozoospermia	5.41	(1.94–15.08)	0.0013
Azoospermia	ref	--	--
**Sertoli Cell Only**			
Yes	0.16	(0.08–0.31)	<0.0001
No	ref	--	--
**Hypospermatogenesis**			
Yes	5.82	(1.61–21.05)	0.0073
No	ref	--	--
**Preoperative Testosterone**			
<300	0.44	(0.21–0.90)	0.0251
≥300	ref	--	--

## Data Availability

Data is publicly unavailable due to institutional privacy restrictions.
